# 125 years of the plague in Brazil: lessons learnt, historical insights and contemporary challenges

**DOI:** 10.1590/0074-02760240220

**Published:** 2025-02-24

**Authors:** Igor Vasconcelos Rocha, Matheus Filgueira Bezerra, Marise Sobreira, Alzira Maria Paiva de Almeida

**Affiliations:** 1 Fundação Oswaldo Cruz-Fiocruz, Instituto Aggeu Magalhães, Departamento de Microbiologia, Recife, PE, Brasil

**Keywords:** plague, Yersinia pestis, public health, epidemics, historical epidemiology

## Abstract

The history of the plague, caused by *Yersinia pestis*, is marked by some of the most devastating pandemics. Its arrival in Brazil on the turn of the 19-20th century led to significant public health challenges and responses. Here, we discuss a comprehensive perspective on the history of the plague in Brazil, emphasising epidemiological trends, public health responses and scientific advances. Understanding the history of the plague in Brazil provides valuable insights into infectious disease control. The study highlights the importance of early detection, robust public health infrastructure, and ongoing research, emphasising the lasting influence of epidemic diseases on society.

The history of the plague is an emblematic example of the enduring impact of infectious diseases on human societies. Caused by the bacterium *Yersinia pestis*, plague is primarily transmitted by infected fleas between wild rodents, eventually reaching humans.^1^ ^,^ ^2^ The plague has been responsible for some of the most devastating pandemics in recorded history, including the Black Death in the 14th century.^1^ The disease manifests in three main primary forms: bubonic, characterised by swollen lymph nodes or buboes; septicaemic, which involves the bloodstream; and pneumonic, affecting the lungs and capable of human-to-human transmission via respiratory droplets.^3^

The arrival of the plague in Brazil at the dawn of the 20th century marked a new chapter in the nation’s public health history.^4^ ^,^ ^5^ The disease first struck the port city of Santos in 1899, spreading rapidly to other major urban centres such as Rio de Janeiro, Salvador and Recife.^5^ The response, led by Dr Oswaldo Cruz, represented a turning point in how Brazil addressed sanitary emergencies, setting a new standard for public health interventions.^6^ These outbreaks prompted an urgent response from both the government and the scientific community, leading to the implementation of quarantine measures, public health campaigns, and the establishment of specialised health institutions.

Over time, the incidence of plague in Brazil declined, thanks to improved public health measures, nevertheless, the disease continued to occur in some rural areas, with sporadic cases.^7^ Contemporary strategies to enhance and optimise plague monitoring and prevention continue to evolve through combining historical experiences and innovative scientific research.

Here, we present a comprehensive perspective on the history of the plague in Brazil, examining its epidemiological trends, the public health response, socioeconomic impacts, diagnosis and scientific advances.

In this article, the historical overview of plague in Brazil derived from the extensive archive from the Brazilian National Plague Reference Service (Serviço de Referência Nacional em Peste - SRP), personal experience from the team and review of historical records, epidemiological studies, and scientific articles. These information sources allowed us to get a clear picture of plague management and public health responses in Brazil.

History and evolution of plague control measures

With the arrival of the plague in Rio de Janeiro, the capital of the country at the time, in 1900, Oswaldo Cruz was tasked by the government to study the epidemic that was ravaging the city.^6^ He conducted extensive research on the microbiology, transmission, symptoms, and pathology of the disease, providing a comprehensive description of the plague.^4^ ^,^ ^6^ Additionally, he oversaw the production of treatment supplies such as anti-plague serum and vaccines, which were manufactured at the Instituto Soroterápico Federal (Federal Serum Institute). This institute later evolved into the current Fundação Oswaldo Cruz.^6^ ^,^ ^8^ ^,^ ^9^

During this period, Oswaldo Cruz coordinated essential public health measures against the plague in Rio de Janeiro, including fumigating ships and sewers and offering rewards for rodent captures.^10^ This response marked a critical inflection point in public health practices in Brazil. Key interventions involved the eradication of fleas using a soap and kerosene mixture, exterminating rats through methods such as boiling water and arsenic bait, constructing sealed buildings, and implementing periodic control cycles within a six-kilometre radius of infected residences.^11^

Altogether, these measures led to the elimination of plague from the port and the urban areas in Brazil. However, the disease was introduced to rural and sylvatic areas, reaching the local autochthonous rodent and fleas’ fauna and establishing several natural plague foci.

The fight against the plague, initially conducted by the State Health Departments, in 1936 became the responsibility of the National Health Department (Departamento Nacional de Saúde - DNS), and in 1941 to the National Plague Service (Serviço Nacional de Peste - SNP). From 1956 onwards, the SNP was incorporated into the National Department of Rural Endemic Diseases (Departamento Nacional de Endemias Rurais - DNERu), which was later abolished, resulting in the creation of the Superintendence of Campaigns (Superintendência de Campanhas de Saúde Pública - SUCAM, 1970-1990). In 1990, SUCAM was dissolved and replaced by the National Health Foundation (Fundação Nacional de Saúde - FUNASA), which in turn was transformed into the Health Surveillance Secretariat (Superintendência de Vigilância em Saúde - SVS) and finally into the current Health and Environmental Surveillance Secretariat (Secretaria de Vigilância em Saúde e Ambiente - SVSA). The plague laboratory at the Aggeu Magalhães Institute, a result of the work of a research group on the plague since 1966, was officially designated as the Brazilian National Plague Reference Service (SRP) in 2002 (Fig. 1).

**Fig. 1:**
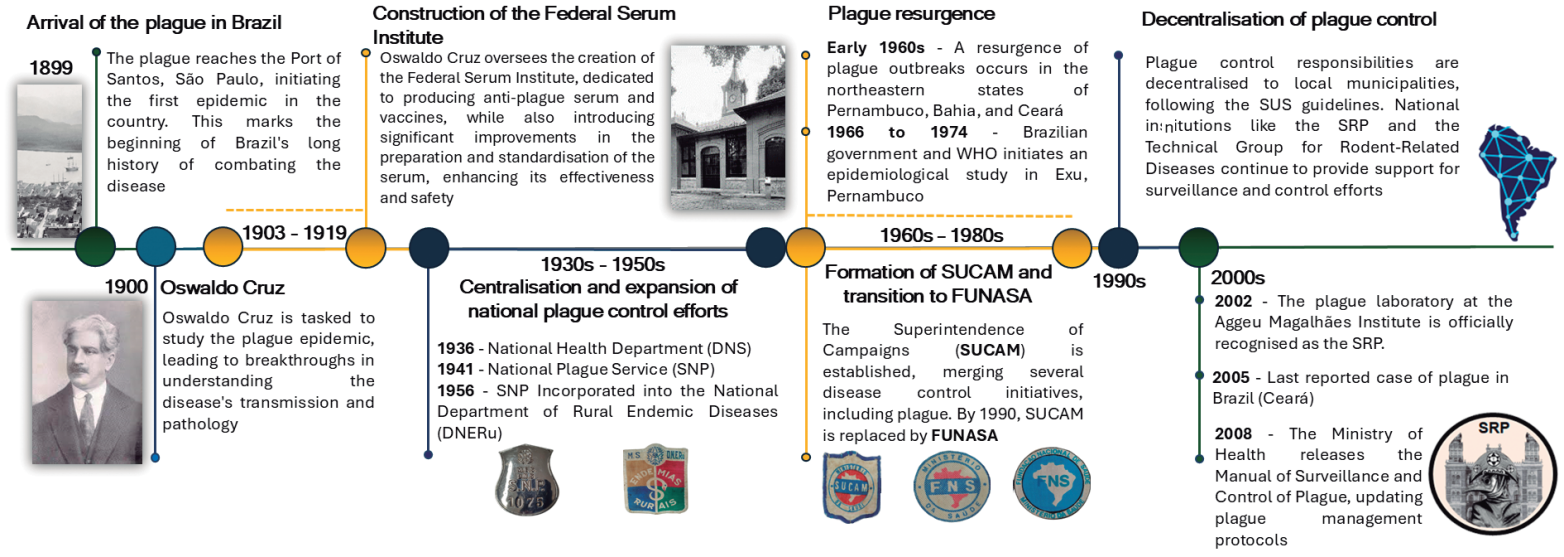
timeline of plague control and institutional developments in Brazil (1899-Present). This timeline illustrates the key events in plague control efforts in Brazil, highlighting the establishment of institutions, public health interventions, and preventive strategies from 1899 to the present.

In line with the guidelines of the Brazilian Ministry of Health (SUS), plague surveillance and control activities, which until then had been a federal government responsibility, were decentralised to the municipal administrations, from the 1990s onwards. The Technical Group for Rodent-Related Diseases from the Ministry of Health (*GT-Roedores*) and the SRP continue to provide support for these activities. Plague cases still occurred at about 20 to 100 cases annually. In the 1950s, the decline in cases and morbidity led to a loss of interest in plague; laboratories were deactivated, and personnel migrated to other programs (universities and clinics). Unfortunately, in the early 1960s, there was a resurgence of plague, particularly in the states of Pernambuco, Bahia, and Ceará.^11^ This prompted the Brazilian government, in collaboration with the World Health Organization (WHO), to establish a program to study the epidemiology of plague. This project was developed from 1966 to 1974 and was based in the municipality of Exu, in the transmission area of the Chapada do Araripe, which was the most active plague area in the country at that time. The results from this project allowed the reorganisation of the plague surveillance and control program with new strategies, such as strengthening active surveillance through regular monitoring of rodent and flea populations in endemic regions, establishing sentinel surveillance sites for early outbreak detection, and enhancing diagnostic capabilities in local laboratories.

At the turn of the 21st century, the behaviour of the zoonosis changed again, with cases declining and infection detected only through serological surveillance in stray dogs, which were adopted as sentinel animals. A technical guideline from the Ministry of Health established the suspension of surveillance through bacterial research in rodents and fleas, as well as the search for antibodies in rodents,^12^ which were considered weak detectors during interepizootic periods.

Therapeutic strategies and diagnostic approaches

Before the advent of the current therapeutic arsenal for treating patients and the availability of the rodenticides and insecticides effective against rodent/hosts and flea/vectors the resources for the plague control were limited and mostly based on non-rigorous evidence.^13^

Chemoprophylaxis for those exposed to the disease initially relied on passive immunisation with anti-plague serum, which was eventually replaced by sulphonamides such as sulfanilamide, sulfathiazole, and sulfadiazine.^3^ Streptomycin became known as the standard treatment for the plague, significantly reducing mortality rates. More recently, newer antibiotics, including aminoglycosides (gentamicin and amikacin), fluoroquinolones (ciprofloxacin, levofloxacin, and ofloxacin), tetracyclines (doxycycline), chloramphenicol, and sulfonamides, have been employed.^3^

The anti-plague serum, first detailed by Vasconcellos in the Memórias do Instituto Oswaldo Cruz journal in 1909, was widely used until the introduction of antibiotics.^14^ Its use as treatment and prophylaxis for plague in Brazil and globally continued into the mid-20th century, primarily until the 1950s.^14^ ^,^ ^15^ Initially, serum therapy was often combined with sulpha drugs, among the first effective treatments for bacterial infections.^14^ ^,^ ^15^ However, the development and widespread adoption of specific antibiotics^3^ led to a gradual decline in serum use.^14^ ^,^ ^15^ ^,^ ^16^ As these antibiotics became the standard of care,^3^ ^,^ ^17^ reliance on serum therapies diminished significantly, marking a transition to modern treatments.^3^ ^,^ ^14^ ^,^ ^15^ ^,^ ^16^ ^,^ ^17^

A plague vaccine developed by Siebra de Brito from the National Plague Service (SNP), in Minas Gerais, was never extensively used in rural areas and was abandoned with the advent of sulphonamides, which proved to be more effective.^3^ ^,^ ^13^ With the rise of antibiotics, the use of the vaccine was completely abolished, and it was no longer produced in Brazil. Before 1940, the SNP used a killed germ vaccine prepared at Oswaldo Cruz Institute. In 1949, the SNP laboratory developed a vaccine using the live EV strain,^15^ which was only used experimentally.

After the 1990s, the production of supplies and the development of new techniques for plague control advanced significantly. The publication of the *Manual of Surveillance and Control of Plague* ^18^ solidified modern approaches to managing the disease in the Brazilian plague areas.

In terms of rodent control, the use of rodenticides has largely been replaced by anti-ratisation measures, which focused on keeping rodents away from human dwellings by removing food sources and shelters rather than attempting to kill the animals directly.

In Brazil, approximately 300 individuals died from the plague in 1900, with total deaths recorded as 199 in 1901, 215 in 1902, 360 in 1903, and 274 in 1904.^19^ Although the plague is currently treated,^(1, 3)^ it still affects about 650 people annually worldwide, resulting in approximately 120 deaths, primarily in Africa, and poses a potential hazard in regions with inadequate sanitation.^19^

The last recorded human case of plague in Brazil occurred in 2005 in Ceará, with previous cases reported in Rio de Janeiro (1967), Alagoas (1973), Piauí (1975), Pernambuco (1982), Minas Gerais (1984), Rio Grande do Norte (1987), Paraíba (1989), Ceará (1997), and Bahia (2000) (data provided by the SRP).

The surveillance of plague requires the use of rapid and efficient diagnostic methods, particularly techniques that are sensitive and specific to prevent the spread of the disease among the human population. The SRP has been actively developing and evaluating bacteriological, serological, and molecular diagnostic methods that can be employed in routine diagnostics, as well as in emergency situations. In addition to these diagnostic advancements, the SRP has been involved in producing key reagents and materials for the study and diagnosis of plague. The following sections will provide detailed descriptions of each of these diagnostic methods and reagents.

Serological, bacteriological, and molecular approaches in plague diagnosis

Historically, the diagnosis of plague has relied on the identification of specific bacterial antigens and the detection of antibodies against these antigens in infected hosts^20^ (Fig. 2). One of the most critical antigens used in plague diagnostics is the F1 antigen, a capsular protein encoded by the *caf1* gene, which is highly immunogenic and specific to *Y. pestis*.^20^ Traditional protocols for detecting plague involve utilising the F1 antigen in haemagglutination assays, where antibodies to the F1 protein are identified in patient samples or animal reservoirs.^21^ ^,^ ^22^

**Fig. 2:**
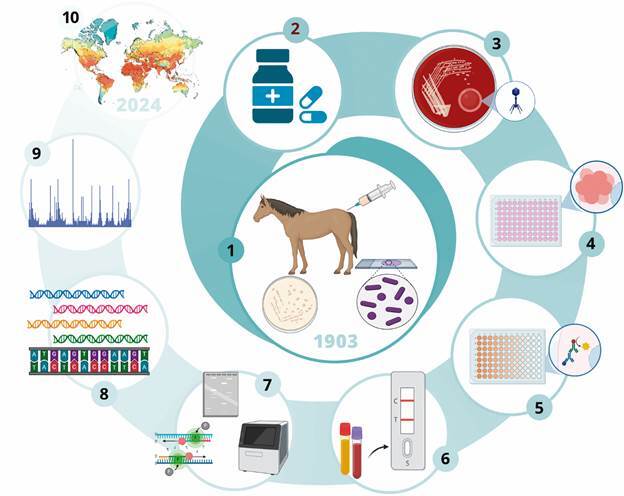
evolution of the plague management: an overview of technological advancements since anti-plague serum production (1903-2024). The scheme illustrates the evolution of plague management, from the early production of anti-plague serum to advanced molecular diagnostics and cutting-edge technologies. The central element (1) represents the production of anti-plague serum, surrounded by key advancements in diagnosis and treatment: (2) antibiotic therapy; (3) bacterial culture; (4) haemagglutination test; (5) ELISA; (6) rapid diagnostic test; (7) PCR methods (multiplex, qPCR, ddPCR); (8) high-throughput sequencing; (9) MALDI-TOF; and (10) eco-epidemiological studies.

The F1 antigen has been a cornerstone in serological tests for plague. The WHO has long recommended detecting anti-F1 antibodies through haemagglutination techniques as part of routine plague surveillance.^23^ However, traditional methods of producing F1 antigen involve culturing large amounts of *Y. pestis*, which must be done under Biosafety Level 3 (BSL-3) conditions due to the pathogenic nature of the bacterium.^24^ Following cultivation, the F1 protein is typically extracted via ammonium sulphate precipitation, a labour-intensive and costly process that demands specialised containment facilities to mitigate biohazard risks.^23^ ^,^ ^25^

To address these challenges and reduce associated risks, recent advancements have focused on using recombinant F1 antigens. Tavares et al.^26^ describes a significant development in this area: the alternative production of recombinant F1 protein (F1-rec) in a non-pathogenic *Escherichia coli* strain without the need for a high-containment infrastructure.^26^

Advancements in serological diagnostics for plague have led to the development of innovative matrices and materials designed to enhance the accuracy and efficiency of diagnostic tests.^27^ ^,^ ^28^ ^,^ ^29^ ^,^ ^30^ ^,^ ^31^

The development of ELISA methods marked a significant improvement, offering faster and more efficient plague detection while maintaining high sensitivity and specificity.^32^ Recent advancements in diagnostics include the development of multi-species and rapid assay technologies.^33^ The multi-species Protein A-ELISA represents a significant advancement, exhibiting superior sensitivity and specificity in detecting anti-F1 antibodies across a broad range of mammalian hosts, including humans.^22^ Additionally, the introduction of a new rapid diagnostic test (RDT) also based on the F1 capsular antigen further enhances diagnostic capabilities.^33^ Complementing the Protein A-ELISA, the RDT provides a practical solution for immediate field-based and point-of-care diagnostics, demonstrating high sensitivity and specificity while addressing the challenges of laboratory infrastructure in low-resource settings.^33^

The bacteriological diagnosis of plague involves several key methods for identifying *Y. pestis*. Bacterioscopy from smears are prepared from clinical samples, fixed, and stained, then examined under a microscope for characteristic features of *Y. pestis*. Gram staining allows for the observation of the typically bipolar Gram-negative bacilli.^18^ Additionally, Congo red staining can be employed to visualise the bacterial capsule.^34^ Although immunofluorescence techniques can enhance the identification of *Y. pestis*, their use has become less frequent due to the development of more efficient methods.^35^

Bacteriological culture remains the gold standard for plague diagnosis.^1^ Over the years, the development of new culture media has significantly improved the isolation and differentiation of *Y. pestis*, especially in challenging conditions such as those encountered in rural landscapes, where environmental contamination from field samples (*e.g.*, rodents and fleas) is common.^36^ ^,^ ^37^ Selective media such as Cefsulodin-Irgasan-Novobiocin Agar (CIN) and Brain heart infusion agar, Irgasan, and Nystatin (BIN) have been developed to address these challenges by inhibiting the growth of contaminating organisms while facilitating the isolation of *Y. pestis*.^36^ ^,^ ^37^ More recently, CYP broth has emerged as a valuable tool in *Y. pestis* culture. This medium builds upon LB broth and is enriched with selective agents and the ferrioxamine E to promote the growth of *Yersinia* species, optimising the growth of *Y. pestis*, even from low-quality or heavily contaminated samples.^38^ The use of selective culture media combined with MALDI-TOF mass spectrometry has significantly improved diagnostic accuracy. When paired with Bruker’s Security-Relevant Library (BSRL), specifically designed for high-risk pathogen identification, MALDI-TOF becomes a powerful tool for identifying *Y. pestis*.^39^

Molecular methods provide rapid results, enabling immediate implementation of control measures and proving particularly effective when samples are not viable for bacteriological testing. The SRP has developed several polymerase chain reaction (PCR)-based assays for plague diagnosis, targeting *Y. pestis* detection from rodents, fleas, and human samples, including multiplex-PCR (M-PCR), nested-PCR, and LAMP.^40^ ^,^ ^41^ Among them, the M-PCR is particularly useful for analysing various biological materials such as blood, bubo aspirates, rodent viscera, and fleas. M-PCR targets key virulence genes of *Y. pestis*, including the plasmid-borne genes *caf1*, *pla*, and *lcrv*, as well as the chromosomal gene *irp2*.^42^ Quantitative PCR (qPCR) has also emerged as a pivotal method in plague diagnostics due to its capacity to quantify pathogen load and detect low infection levels. Recent advancements in qPCR technology have markedly enhanced the limit of detection of molecular methods and has robust effectiveness in detecting *Y. pestis* across a wide range of environmental and clinical samples.^43^ Droplet digital PCR (ddPCR) also represents a significant advancement in plague diagnostics, enhancing the detection of *Y. pestis* in complex samples, improving sensitivity and enabling precise quantification, thereby strengthening plague surveillance and outbreak response efforts.^44^ Additionally, high-throughput sequencing (HTS) has become an invaluable tool in plague diagnostics, enabling comprehensive genomic analyses of *Y. pestis* strains.^45^ HTS facilitates the identification of genetic variations and resistance mechanisms, enhancing our understanding of the epidemiology and evolution of plague, which is crucial for developing targeted public health interventions and improving diagnostic methodologies.^45^

Perspectives

The analysis presented here highlights the multifaceted approaches that have been employed to understand and control plague throughout its history. Historical accounts demonstrate how the emergence and spread of plague have significantly influenced public health policies and scientific research. The evolution of diagnostic techniques, from early methods of plague detection to modern molecular approaches, underscores the progress made in our ability to detect and manage this critical public health issue.

Current research continues to build with advancements in genomics and proteomics, providing deeper insights into *Y. pestis.* Moreover*,* it is important to advance on the comprehension on how environmental conditions such as rainfall, vegetation, and altitude, as well as host and vector ecology interplay in the risk of new plague outbreaks in its natural foci.^44^ Modelling outbreak risk based on these conditions will allow for more precise public health and capacity-building interventions, towards more efficient surveillance efforts.

Moreover, the historical perspective on plague management strategies reveals the importance of continuous adaptation to emerging threats. The historical lessons learned from past outbreaks should inform current public health strategies and preparedness plans. This includes revisiting and updating surveillance systems, as well as ensuring robust response mechanisms to handle potential re-emergence of plague in areas where it was previously under control.

While significant strides have been made in the study and management of plague, ongoing research and technological innovations hold the promise of further advancements. A comprehensive approach that combines historical insights with modern scientific methods would be crucial in addressing the challenges posed by plague and *Y. pestis*.

## References

[1] Barbieri R, Signoli M, Chevé D, Costedoat C, Tzortzis S, Aboudharam G (2021). Yersinia pestis the natural history of Plague. Clin Microbiol Rev.

[2] Mahmoudi A, Krystufek B, Sludsky A, Schmid BV, Almeida AMP, Lei X (2021). Plague reservoir species throughout the world. Integr Zoology.

[3] Sebbane F, Lemaître N (2021). Antibiotic therapy of Plague a review. Biomolecules.

[4] Nascimento DR (2011). La llegada de la peste al Estado de São Paulo en 1899. Dynamis.

[5] Tavares C, Aragão AI, Leal NC, Leal-Balbino TC, Oliveira MBM, Gonçalves-Ferreira GMO (2012). Plague in Brazil from now and then. Adv Exp Med Biol.

[6] Cukierman HL (1998). Journey(s) to Santos. Hist Cienc Saude-Manguinhos.

[7] Fernandes DLRS, Bezerra MF, Bezerra MS, Leal NC, Souza-Reis CR, Almeida AMP (2021). Rodent hosts and flea vectors in Brazilian plague foci a review. Integr Zool.

[8] Cruz OG (1901). A vaccinação anti-pestosa - Trabalho do Instituto Sôrotherápico Federal do Rio de Janeiro (Instituto de Manguinhos).

[9] Cruz OG (1906). Peste.

[10] Bezerra MF, Almeida AMP (2022). Important infectious diseases in Latin America and the Caribbean: Plague. Springer.

[11] Fernandes DLRS, Gomes ECS, Bezerra MF, Guimarães RJPS, Almeida AMP (2021). Spatiotemporal analysis of bubonic plague in Pernambuco, northeast of Brazil case study in the municipality of Exu. PLoS One.

[12] SVS (2007). Nota Técnica 001. Programa de Controle da Peste.

[13] Freitas CA (1988). Histórias da Peste e de outras endemias.

[14] Vasconcellos F (1909). O sôro anti-pestozo. Mem Inst Oswaldo Cruz.

[15] Pollitzer R (1954). Plague. World Health Organization monograph series. World Health Organization.

[16] Moll AA, O'Leary SB (1940). Plague in the Americas an historical and quasi-epidemiological survey. Pan Am Health Org.

[17] Godfred-Cato S, Cooley KM, Fleck-Derderian S, Becksted HA, Russel Z, Meaney-Delman D (2020). Treatment of human plague a systematic review of published aggregate data on antimicrobial efficacy, 1939-2019. Clin Infect Dis.

[18] MS/SVS/DVE - Ministério da Saúde/Secretaria de Vigilância em Saúde/Departamento de Vigilância Epidemiológica (2008). Manual de vigilância e controle da Peste.

[19] Fioravanti C (2020). Guerra à Peste. Pesq Fapesp.

[20] Rajerison M, Dartevelle S, Ralafiarisoa LA, Bitam I, Tuyet DTN, Andrianaivoarimanana V (2009). Development and evaluation of two simple, rapid immunochromatographic tests for the detection of Yersinia pestis antibodies in humans and reservoirs. PLoS Negl Trop Dis.

[21] Balakrishna K, Tuteja U, Murali HS, Batra HV (2014). Application of r-PFE hyperimmune sera for concurrent detection of Bacillus anthracis, Yersinia pestis and staphylococcal enterotoxin B. J Appl Microbiol.

[22] Bezerra MF, Xavier CC, Almeida AM, Reis CRS (2022). Evaluation of a multi-species Protein A-ELISA assay for plague serologic diagnosis in humans and other mammal hosts. PLoS Negl Trop Dis.

[23] WHO - World Health Organization (1970). Technical report series WHO Expert Committee on Plague. Fourth Report. World Health Organization Bulletin.

[24] Brady A, Tomaszewski M, Garrison TM, Lawrenz MB (2024). Approaches for the inactivation of Yersinia pestis. Appl Biosaf.

[25] Chu M (2000). Laboratory manual of plague diagnostic tests. Centers for Disease Control and Prevention/World Health Organization.

[26] Tavares DHC, Bezerra MF, Magalhães FB, Cavalcanti TYVL, Xavier CC, Leal NC (2020). A new recombinant F1 antigen as a cost and time-effective tool for plague diagnosis. J Microbiol Methods.

[27] Almeida AMP, Leal NC, Brasil DP, Almeida CR (1987). Estudo comparativo entre as técnicas de hemaglutinação e aglutinação bacteriana no diagnóstico da peste humana. Rev Bras Microbiol.

[28] Araujo AM, Petribú ATS, Barbosa GHTS, Diniz JRP, Almeida AMP, Azevedo WM (1996). The use of polyvinyl alcohol glutaraldehyde as solid-phase in ELISA for plague. Mem Inst Oswaldo Cruz.

[29] Barbosa GHTS, Santana EM, Almeida AMP, Araujo AM, Fatibello-Filho O, Carvalho-Júnior LB (2000). The use of filter paper plasticized with polyvinyl alcohol-glutaraldehyde in ELISA. Braz J Med Biol Res.

[30] Montenegro SML, Almeida AMP, Carvalho AB, Carvalho-Júnior LB (1991). The use of dacron plates for DOT enzyme-linked immunosorbent assay (DOT-ELISA). Mem Inst Oswaldo Cruz.

[31] Almeida AMP, Ferreira LCS (1992). Evaluation of three serological tests for the detection of human plague in northeast Brazil. Mem Inst Oswaldo Cruz.

[32] Araujo AM, Petribú ATS, Barbosa GHTS, Diniz JRP, Almeida AMP, Carvalho-Júnior LB (1998). Rapid Elisa for Plague. Mem Inst Oswaldo Cruz.

[33] Bezerra MF, Santos WJT, Rocha IV, Nadaes NR, Dantas-Torres F, Sales KGS (2022). Performance assessment of a new indirect rapid diagnostic test for plague detection in humans and other mammalian hosts. Acta Trop.

[34] Rocha IV, Bezerra MF, Sobreira M, Leal NC, Almeida AMP (2024). Lyophilization for bacteria preservation a promising approach for Yersinia pestis strains from an unique collection in Brazil (Fiocruz-CYP). Anton Van Leeuwenhoek.

[35] Phillips AP, Morris BC, Hall D, Glenister M, Williams JE (1988). Identification of encapsulated and non-encapsulated Yersinia pestis by immunofluorescence tests using polyclonal and monoclonal antibodies. Epidemiol Infect.

[36] Aftalion M, Aloni-Grinstein R, Andrianaivoarimanana V, Iharisoa AL, Shmaya S, Gur D (2021). Improved selective BIN agar for a better rate of Yersinia pestis isolation from primary clinical specimens in suspected Madagascar plague cases. J Clin Microbiol.

[37] Sarovich DS, Colman RE, Price EP, Chung WK, Lee J, Schupp JM (2010). Selective isolation of Yersinia pestis from plague-infected fleas. J Microbiol Methods.

[38] Rocha IV, Andrade CAN, Sobreira M, Leal NC, Almeida AMP, Bezerra MF (2023). CYP broth a tool for Yersinia pestis isolation in ancient culture collections and field samples. Appl Microbiol Biotechnol.

[39] Feng B, Shi L, Zhang H, Shi H, Ding C, Wang P (2021). Effective discrimination of Yersinia pestis and Yersinia pseudotuberculosis by MALDI-TOF MS using multivariate analysis. Talanta.

[40] Souza G, Almeida A, Farias A, Leal N, Abath F (2007). Development and evaluation of a single tube nested PCR based approach (STNPCR) for the diagnosis of plague. Adv Exp Med Biol.

[41] Lira-Nunes M, Mendes-Marques CL, Almeida AMP, Leal NC (2014). The development of a loop-mediated isothermal amplification (LAMP) procedure for plague diagnostic. Am J Anal Chem.

[42] Leal NC, Almeida AMP (1999). Diagnosis of plague and identification of virulence markers in Yersinia pestis by multiplex-PCR. Rev Inst Med Trop São Paulo.

[43] Bai Y, Motin V, Enscore RE, Osikowicz L, Rosales Rizzo M, Hojgaard A (2020). Pentaplex real-time PCR for differential detection of Yersinia pestis and Y pseudotuberculosis and application for testing fleas collected during plague epizootics. Microbiologyopen.

[44] Zhao Y, Yan Z, Song K, Li Y, Shen L, Cui Y (2024). Development and evaluation of a multi-target droplet digital PCR assay for highly sensitive and specific detection of Yersinia pestis. PLoS Negl Trop Dis.

[45] Bezerra MF, Fernandes DLRS, Rocha IV, Pitta JLLP, Freitas NDA, Oliveira ALS (2024). Ecologic, geoclimatic, and genomic factors modulating plague epidemics in primary natural focus, Brazil. Emerg Infect Dis.

